# Differential Contributions of Specimen Types, Culturing, and 16S rRNA Sequencing in Diagnosis of Prosthetic Joint Infections

**DOI:** 10.1128/JCM.01351-17

**Published:** 2018-04-25

**Authors:** Lone Heimann Larsen, Vesal Khalid, Yijuan Xu, Trine Rolighed Thomsen, Henrik C. Schønheyder

**Affiliations:** a Department of Clinical Microbiology, Aalborg University Hospital, Aalborg, Denmark; b Center for Microbial Communities, Department of Chemistry and Bioscience, Aalborg University, Aalborg, Denmark; c Department of Orthopaedic Surgery, Aalborg University Hospital, Aalborg, Denmark; d Biotech, Danish Technological Institute, Aarhus, Denmark; e Department of Clinical Medicine, Aalborg University, Aalborg, Denmark; Department of Orthopedic Surgery, Aalborg University Hospital, Aalborg, Denmark; Department of Nuclear Medicine, Aalborg University Hospital, Aalborg, Denmark; Department of Infectious Diseases, Aalborg University Hospital, Aalborg, Denmark; Danish Technological Institute, Biotech, Aarhus, Denmark; Department of Chemistry and Bioscience, Aalborg University, Aalborg, Denmark; Department of Health Science and Technology, Faculty of Medicine, Aalborg University; Mayo Clinic

**Keywords:** joint prosthesis, infection, biofilm, diagnosis (microbiology), RNA, ribosomal, 16S, 16S RNA, biofilms, diagnostics, joint infections, prospective clinical study, prosthesis infections

## Abstract

Prosthetic joint failure is mainly caused by infection, aseptic failure (AF), and mechanical problems. Infection detection has been improved with modified culture methods and molecular diagnostics. However, comparisons between modified and conventional microbiology methods are difficult due to variations in specimen sampling. In this prospective, multidisciplinary study of hip or knee prosthetic failures, we assessed the contributions of different specimen types, extended culture incubations, and 16S rRNA sequencing for diagnosing prosthetic joint infections (PJI). Project specimens included joint fluid (JF), bone biopsy specimens (BB), soft-tissue biopsy specimens (STB), and swabs (SW) from the prosthesis, collected *in situ*, and sonication fluid collected from prosthetic components (PC). Specimens were cultured for 6 (conventional) or 14 days, and 16S rRNA sequencing was performed at study completion. Of the 156 patients enrolled, 111 underwent 114 surgical revisions (cases) due to indications of either PJI (*n* = 43) or AF (*n* = 71). Conventional tissue biopsy cultures confirmed PJI in 28/43 (65%) cases and refuted AF in 3/71 (4%) cases; one case was not evaluable. Based on these results, minor diagnostic adjustments were made. Fourteen-day cultures of JF, STB, and PC specimens confirmed PJI in 39/42 (93%) cases, and 16S rRNA sequencing confirmed PJI in 33/42 (83%) cases. One PJI case was confirmed with 16S rRNA sequencing alone and five with cultures of project specimens alone. These findings indicated that JF, STB, and PC specimen cultures qualified as an optimal diagnostic set. The contribution of sequencing to diagnosis of PJI may depend on patient selection; this hypothesis requires further investigation.

## INTRODUCTION

The issue of which specimens would be optimal to acquire during revision surgery for patients suspected of prosthetic joint infection (PJI) has been repeatedly contested. In Scandinavian countries, since the early 1980s, multiple periprosthetic soft-tissue biopsy specimens (STB) have been used extensively (1), and these have become standard for diagnosing PJIs. However, the number of culture-positive biopsy specimens needed to diagnose a PJI has varied (2). In 1998, a prospective study by Atkins et al. (3) reported a high positive predictive value for two or more culture-positive biopsy specimens, and this finding was later confirmed in another study (4). In the late 1990s, biofilms were identified on prosthetic elements from infected hip joints (5). This finding led to new diagnostic options, including sonicating the removed prosthesis and culturing the sonication fluid (6, 7).

Two recently published sets of European and American guidelines recommended collecting two to six intraoperative tissue biopsy specimens during surgery and submitting them to culture. Infections would be indicated when two or more biopsy specimens were positive for the same microorganism. As an adjunct, an examination of prosthetic components (PCs) was recommended for diagnostic work-ups (8, 9).

Currently, culture assays remain the standard for ascertaining a PJI diagnosis; however, since the late 1990s, molecular diagnostics have gained ground, particularly methods that target 16S rRNA with either multiplex PCR or sequencing strategies (10–12). Newer studies have investigated the diagnostic accuracy of combining molecular methods and specimen types, but different studies have reported varying sensitivity (13, 14). A growing number of in-house molecular tests have emphasized the need for independent studies of clinical utility (15, 16). In addition, although several specimen types have been studied extensively (6, 14, 17–23), only a few studies had a prospective design that implemented criteria for patient selection, sampling of multiple specimen types, and optimized culture methods (24).

Recently, a prospective Danish study enrolled patients with a painful or dysfunctional hip or knee prosthetic joint. This study provided an opportunity for standardized sampling of different specimen types and application of both culturing and 16S rRNA sequencing for diagnosing PJI. The primary goal was to define an optimal specimen set for diagnosing PJIs; the secondary goal was to define the utility of 16S rRNA sequencing in microbial diagnostics.

(Preliminary results were presented at the European Congress of Clinical Microbiology and Infectious Diseases [ECCMID] in 2014 [25] and 2015 [26] and at the 33rd Annual Meeting of the European Bone & Joint Infection Society [EBJIS] in 2014 [27]).

## MATERIALS AND METHODS

### Diagnostic algorithm.

The diagnostic algorithm differed from the standard care mainly in two aspects. First, the algorithm included an option for radionuclide imaging, and when a hot spot was revealed, it was followed by a biopsy procedure (27). Second, the algorithm included an extensive work-up of the specimens obtained during surgical revision (details given below). Deviations from the algorithm were accepted based on preferences of the patient or surgeon. For example, a joint puncture was not recommended as a primary diagnostic step, because it might interfere with radionuclide imaging; however, it was permissible when deemed necessary by the surgeon. Radionuclide imaging was performed in a subset of patients, and images were evaluated by a multidisciplinary specialist team. However, radionuclide imaging did not provide independent diagnostic evidence, and the images were not considered in this study.

### Specimens.

Joint fluid was collected with a separate diagnostic procedure at the surgeon's discretion (see above). Also, prior to any joint capsule incision during revision surgery, sampling was obligatory. The sets of five STBs (5-STB) were collected from a single periprosthetic site, according to local guidelines and as described previously by Kamme and Lindberg (1). The project specimens comprised three periprosthetic STBs, three swabs (SWs) (ESwab, Copan, Italy), three periprosthetic bone biopsy specimens (BBs), and any removed PCs. The STBs were acquired from a periprosthetic site (preferably with signs of infection), the prosthesis SWs were taken from the interface between bone and the prosthesis, and the BBs were likewise collected from bone in contact with the prosthesis. Each specimen was placed in a separate sterile tube provided in the prepacked box (28). This box was used for transporting the specimens at ambient temperature to the laboratory. Most specimens were processed within 2 h after the revision (a delay of up to 24 h was permitted). Specimens from acute surgeries performed outside business hours were maintained at 4°C overnight.

### Specimen processing. (i) Bacteriological culture.

Procedures for specimen assays, including light microscopy, culture media, and incubations, are described in detail in Text S1 in the supplemental material. Components of the prosthesis were immersed in sterile 1× phosphate-buffered saline (PBS) buffer, pH 7.4, and then vortexed and sonicated, essentially as described elsewhere (24). The sonication fluid was cultured, and the number of CFU was estimated semiquantitatively and summed over all components without a predefined cutoff value. Likewise, BBs were submitted to vortexing and sonication before culturing. Prosthetic SWs were vortexed in the transport medium (Copan, Italy) and the liquid phase was cultured (Copan). All cultures were incubated for 14 days; cultures on aerobic media were examined on days 1, 2, 4, 6, 10, and 14, and cultures on anaerobic media were examined on days 2, 4, 6, 10, and 14.

### (ii) 16S rRNA sequencing.

Specimens were prepared for molecular analysis by vortexing for 30 s, and then they were stored in minimum 10% glycerol at −80°C until batch-wise processing and analysis.

Molecular analyses were performed in a standardized manner for the five specimen types. STB, BB, joint fluid (JF), and sonication fluid from the PCs were processed without any additional mechanical preparation. Prosthetic SWs were vortexed in the transport medium, and the liquid phase was used for molecular analysis.

One STB per patient was incubated with Proteinase K until it was completely dissolved. DNA was extracted from all specimen types with MolYsis Complete5 (Molzym, Germany) according to the manufacturer's protocol. The other two STB were kept in reserve. A negative control (no sample included) and a positive control (either Escherichia coli or Streptococcus pyogenes) was included for every 22 specimens. All DNA extracts were screened for the presence of bacterial DNA by amplifying the full-length 16S rRNA with endpoint PCR (29). For all specimens that showed the presence of bacterial DNA and for the positive and negative controls, we created 16S rRNA amplicon sequencing libraries of the v1-v3 region. These libraries were constructed according to a modified in-house protocol (30) with specific primers (forward primer, 5′-AGAGTTTGATCCTGGCTCAG-3′; reverse primer, 5′-ATTACCGCGGCTGCTGG-3′; ∼488 bp), *Taq* polymerase, and 10× Key buffer (VWR, Bie & Berntsen, Søborg, Denmark) with the MiSeq system (Illumina, USA).

The sequences were clustered into operational taxonomic units (OTU), at both 97% and 99% similarities, to obtain separation at both the genus and species levels. We then performed sequence alignments in the MIDAS database (30). We used the BLAST tool in the NCBI database (National Center for Biotechnology) to search for matches to sequences that were not identified or to obtain more detailed taxonomic information. All of the control sequences used in DNA extraction and negative PCR assays were sequenced to enable background subtraction in sequencing (see Text S3 for details).

The molecular specimens were analyzed batch-wise after patient inclusion had been completed. Therefore, the results did not affect the recorded diagnosis or treatment of patients.

### Data analysis.

A subgroup of patients had undergone a diagnostic procedure before revision. Some received a joint puncture (*n* = 31), others received a biopsy procedure guided by radionuclide imaging (*n* = 10), and others received a second surgical procedure (*n* = 4). Diagnostic biopsy procedures and joint punctures performed before revision were not included in our data analysis.

Cases were initially grouped according to the working diagnosis (i.e., the surgeon's initial assessment based on the MSIS criteria [31]). Later, they were reevaluated according to the culture report on the 5-STB set on day 6. Infections were corroborated when ≥3 culture-positive STBs exhibited identical bacterial isolates. Cases with ≤2 positive STBs on day 6 were further evaluated based on cultures of project specimens. On day 14, the final assessment was made, based on the results from all specimen types.

Likelihood ratios of positive and negative results (LR+ and LR−) were calculated for each combination of specimen type and method (32, 33). Likelihood ratios were chosen because LR+ and LR− are less influenced by prevalence of the target condition than other measures of accuracy. LR+ is the ratio of the proportion of PJI patients with a positive result to the proportion of AF patients with a positive result. LR− is the ratio of the proportion of PJI patients with a negative result to the proportion of AF patients with a negative result (Table 1). Calculations of LR with 95% confidence limits were carried out at the VassarStats Website for Statistical Computation (Clinical calculator 1 at http://vassarstats.net/).

## RESULTS

### Study design.

The prospective cohort study included patients referred due to pain in a hip or knee arthroplasty and a possible PJI. Ethical approval was obtained from the Regional Committee on Health Research Ethics for the Northern Denmark Region (N-20110022). The study was conducted in the Northern Denmark Region between December 2011 and February 2014. All residents in Denmark have unfettered access to primary care and public hospitals. Patients waiting for an arthroplasty have a right to seek a private clinic, but all complications are treated in public hospitals. A unique personal identification number is used for all health records, and it provides a key that links all health data on a given individual.

Patients with prosthesis-related problems in hips and knees were included after obtaining informed consent. Patients with fractures and luxation in hip joints were not eligible. The initial assessment included patient history, clinical examination, blood biochemistry (white leukocyte count and C-reactive protein assessments were obligatory), and a standard X-ray evaluated based on criteria set by the Musculoskeletal Infection Society (MSIS) in 2011 (31).

A prepacked box with all the necessary tools, containers, and transport media was provided for joint revisions (and another one for joint punctures) to ensure secure individual handling of multiple specimens, as described in detail elsewhere (25, 28). During revision surgery a set of five STBs (5-STB) was collected for culture, in compliance with guidelines for PJI diagnosis in the Northern Denmark Region (1, 2). The following project specimens were obtained in triplicate: joint fluid (JF), STB, prosthesis swabs (SW), and bone biopsy specimens (BB). A separate container was provided for any PCs removed. The rationale for triplicates was primarily to perfect various techniques. Special clinical conditions (e.g., osteoporosis) might have influenced the completeness of the specimen sets (missing specimens are indicated in Table S2 in the supplemental material).

The surgeon's diagnosis took into account any intraoperative observations and a culture report on the 5-STB set, which was incubated for 6 days, according to standard practice (1). According to the study protocol, clinical microbiologists evaluated any additional isolates during extended incubation from both 5-STB and project specimens. The surgeons had agreed to be notified only if such findings might have an impact on the choice of antibiotic treatment.

### Patient groups.

**FIG 1:**
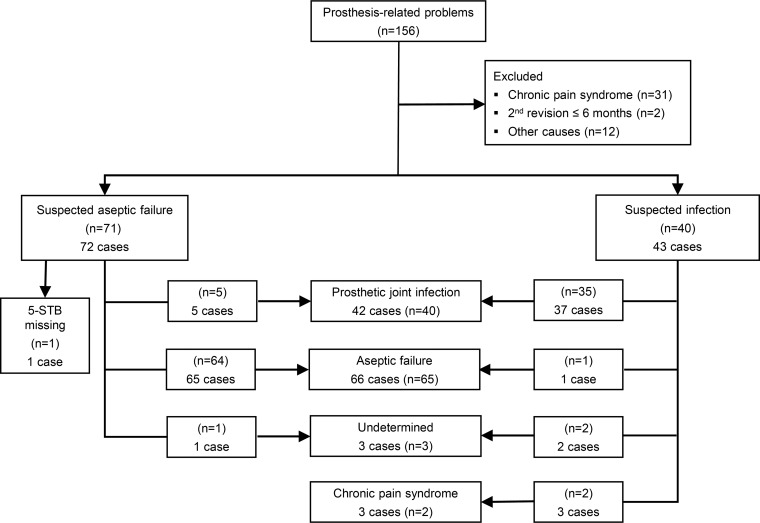
Flow diagram shows the patient selection and allocations. Numbers in brackets refer to patients, but numbers of cases are also indicated due to some patients being admitted more than once.

A total of 156 patients were included (Fig. 1), and 111 underwent one or more revision surgeries. Four patients were included twice, with two revisions conducted at least 6 months apart. Of these, one patient underwent revisions of two arthroplasties for aseptic failure (AF), defined as a prosthetic failure that was deemed unlikely to be associated with an infection. The other three patients underwent two revisions for PJIs; in one patient, two different joints were revised, and in two patients, the same joint required two revisions.

One patient suspected of AF was excluded because the 5-STB set was not collected. Thus, 114 cases were available for evaluation, including 43 cases of revision surgery for PJI and 71 cases of revision surgery for AF (for details, see below). Revisions for PJI were carried out within 12 weeks of the primary surgery in four cases, within 13 to 24 weeks in four other cases, and after 24 weeks in 35 cases.

Among the remaining patients, 31 were diagnosed with a chronic pain condition, and a new surgical procedure was not recommended. Ten patients were diagnosed conclusively after an initial joint puncture.

### Procedures and specimens.

Table S2 provides an overview of completeness of samples and positive results. Of the 43 cases undergoing revision surgery for PJI, 28 (65%) had a positive 5-STB set, defined as at least three positive biopsy specimens in the set of five (Fig. 1). Of the 71 cases undergoing revision for AF, three (4%) had a positive 5-STB set. Thus, 31 cases fulfilled the criterion of a positive 5-STB set (1), and 60 cases had entirely negative 5-STB sets.

Fourteen cases had a working diagnosis of PJI and a negative 5-STB set. Of these, six were diagnosed with PJI based on culture-positive project specimens, three were diagnosed with PJI based entirely on clinical findings (one was supported by 16S rRNA sequencing), one was diagnosed with AF (all specimens were negative), three were diagnosed with a chronic pain condition, and two had undetermined diagnoses (clinical and diagnostic results were inconclusive).

Of the 71 cases with an initial diagnosis of AF, eight had 1 to 2 positive biopsy specimens in the 5-STB set. Of these eight, six had entirely negative project specimens and two had positive project specimens and thus were reallocated into the PJI diagnosis group. One case with an entirely negative 5-STB set was grouped as undetermined based on MSIS criteria (31).

In total, 42 cases were finally diagnosed with PJI, based on MSIS criteria, after diagnostic sampling. Another 66 cases were definitively diagnosed with AF, three cases remained undetermined, and three were diagnosed with chronic pain.

16S rRNA sequencing was performed in all but two cases. Sequencing indicated infections in 83% (33/40) of the group finally diagnosed with PJI; one of these had both a negative 5-STB set and culture-negative project specimens (the sequencing revealed Streptococcus spp.; data not shown). One case in the group diagnosed with AF was positive for an infection (the sequencing revealed Finegoldia magna; data not shown).

### Diagnostic performance.

Accuracy of each combination of specimen type and method (culturing or 16S rRNA sequencing) was evaluated for diagnosis of PJI. The likelihood ratio for a positive result (LR+) and the likelihood ratio for a negative result (LR−) were calculated (Table 1).

**TABLE 1:** Diagnostic accuracy measured by likelihood ratios

Specimen,^*c*^ culture duration (days) or 16S rRNA sequencing	No. of cases with a positive test/no. of cases tested		LR^*a*^	
	PJI (*n* = 42)	AF (*n* = 66)	Positive (95% CI)	Negative (95% CI)
5-STB, 6	31/42	0/66	Infinity^*b*^	0.3 (0.2–0.4)
5-STB, 14	37/42	0/66	Infinity	0.1 (0.05–0.3)
JF, 6	26/39	1/57	38 (5–269)	0.3 (0.2–0.5)
JF, 14	34/39	2/56	24 (6–96)	0.1 (0.06–0.3)
PC, 6	24/37	1/60	39 (5–276)	0.4 (0.2–0.6)
PC, 14	33/37	5/60	11 (5–25)	0.1 (0.05–0.3)
SW (*in situ*), 6	9/32	0/59	Infinity	0.7 (0.6–0.9)
SW (*in situ*), 14	16/32	2/59	15 (4–60)	0.5 (0.4–0.7)
BB, 6	9/32	0/54	Infinity	0.8 (0.7–0.9)
BB, 14	13/32	1/54	22 (3–160)	0.6 (0.5–0.8)
Periprosthetic tissue, 16S rRNA sequencing	8/32	0/53	Infinity	0.8 (0.6–0.9)
Synovial fluid, 16S rRNA sequencing	25/35	1/65	46 (7–328)	0.3 (0.2–0.5)
PC, 16S rRNA sequencing	32/37	1/64	55 (8–389)	0.1 (0.06–0.3)
SW (*in situ*), 16S rRNA sequencing	15/34	4/52	6 (2–16)	0.6 (0.4–0.8)
BB, 16S rRNA sequencing	4/29	2/47	3 (0.6–17)	0.9 (0.8–1.0)

a Positive describes how probability of infection shifts with positive results, and negative describes how probability of infection wanes with negative results. CI, confidence interval.

b An incalculably large number.

c For abbreviations of specimen types, see the text.

### Antibiotics before surgery and 16S rRNA sequencing.

Antibiotic treatment had been administered before surgery in 24 cases. Of these, three cases were in the AF group, and those cultures and 16S rRNA sequencing results were negative. The other 21 cases were in the PJI group. Of these, two showed positive results only with sequencing (Streptococcus spp.); one was definitively diagnosed as a culture-negative infection, and one remained undetermined. The remaining 19 PJI cases showed concordant positive results from cultures and sequencing.

### Infecting microorganisms.

The microbial diversity of PJIs is summarized in Table 2 (for further details, see Table S2). The culture-positive PJI cases showed the following polymicrobial infections (28%; 11/39) based on culture results: two cases of Staphylococcus aureus and coagulase-negative Staphylococcus spp. (CoNS), one case of Streptococcus sp. and Corynebacterium sp., one case of CoNS and Propionibacterium acnes, one case of CoNS and Corynebacterium sp., three cases of different combinations of CoNS species, and four cases of more than three bacterial species. The most prevalent CoNS species were Staphylococcus epidermidis, Staphylococcus capitis, and Staphylococcus lugdunensis. The four patients with early PJI (infections within 12 weeks of the prosthesis insertion) had the following infecting microorganisms: S. aureus, polymicrobial (CoNS, Corynebacterium sp., and Streptococcus sp.), Enterococcus faecalis, and Salmonella enterica serovar Dublin.

**TABLE 2:** Infecting microorganisms in prosthetic joint infections in hips and knees

Microorganism	No. of organisms found in culture^*a*^ (*n* = 39)		No. of organisms determined by 16S rRNA sequencing (*n* = 33)
	Total	Subgroup	
Polymicrobial	11		5
Staphylococcus spp.	14		16
S. aureus		7	
S. epidermidis		2	
S. lugdunensis		3	
S. capitis		1	
CoNS, others		1	
Streptococcus spp.	4		6
Hemolytic streptococcus group C^*b*^		2	
Hemolytic streptococcus group G^*b*^		2	
Enterococcus faecalis	3		2
Escherichia coli	2		
Pseudomonas aeruginosa	1		
Salmonella serovar Dublin	1		1
Propionibacterium spp.	2		2
P. acnes		2	
Finegoldia magna	1		1

a Culturing showed positive results in 39 cases, and sequencing showed positive results in 33 cases. Due to the limited resolution in the 16S rRNA sequencing results, it was not possible to differentiate between species within the genera Staphylococcus, Streptococcus, and Propionibacterium.

b The hemolytic streptococci all were identified in culture as Streptococcus dysgalactiae.

Five patients with an initial diagnosis of AF had the following infecting microorganisms: CoNS (*n* = 3), E. faecalis (*n* = 1), and P. acnes (*n* = 1).

With the exception of the discordant results mentioned earlier, the sequencing results corroborated the culture results and also included additional, uncultivated species. This finding resulted in the discovery of polymicrobial infections. We found five polymicrobial (5/33; 15%) cases: one displayed Escherichia coli in culture plus S. aureus sequences, one displayed E. faecalis in culture plus Finegoldia magna sequences, one displayed several different species in culture, which were corroborated with sequencing, one displayed Staphylococcus sp. and Corynebacterium sp., and one displayed a combination of Staphylococcus spp.; all confirmed the results obtained in cultures. Due to our limited resolution of the v1-v3 region, the 16S rRNA sequencing could not distinguish between Staphylococcus spp. and Streptococcus spp. at the species level.

Five specimen sets were entirely negative based on molecular methods, but some specimens were positive with culturing methods. A semiquantitative assessment of the sonication fluid of the PCs revealed fewer than 200 CFU/ml in four monomicrobial cultures (E. coli, P. acnes, CoNS, and Pseudomonas aeruginosa). The fifth culture was positive for Enterobacter sp., with ∼5 ×10^3^ CFU/ml (data not shown). The cases with P. aeruginosa and Enterobacter sp. were in the group of suspected PJIs; the other three cases were suspected AFs. Two of these were reclassified as PJIs and one remained undetermined.

### Incubation period for project specimens.

**FIG 2:**
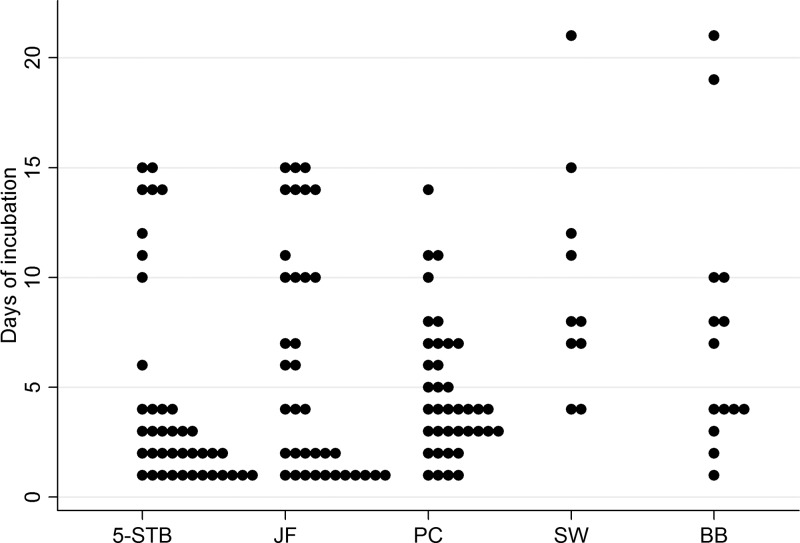
Days of incubation until growth by type of specimen (5-STB, five soft-tissue biopsy specimens; JF, joint fluid; PC, prosthetic components; SW, swabs from the prosthesis, collected *in situ*; BB, bone biopsy specimen). Incubation was planned for 14 days but occasionally was extended due to weekends and holidays.

**FIG 3:**
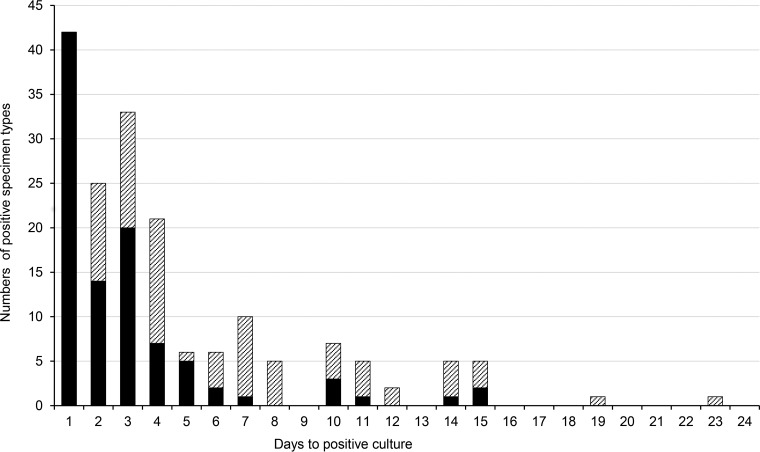
Days to positive culture for the first specimen type (black columns) and subsequent specimen types (hatched).

Within the group of cases with positive cultures, eight did not have positive 5-STB sets on day six, but five of those eight had positive cultures of PCs and JF within this time period (Fig. 2). An extension of the incubation period to not less than 14 days resulted in three additional cases with positive cultures, two in the group suspected of PJI and one in the group suspected of AF (Fig. 3). All specimen types were represented among the late positive cultures (Fig. 2). The infecting microorganisms were S. aureus, S. epidermidis, and P. acnes. Due to the recording method, it was only possible to distinguish between detection of culture growth before or after day six. In no instance was it necessary to modify antibiotic therapy due to late growth of additional bacterial species.

### Contribution of positive cultures by specimen type.

**FIG 4:**
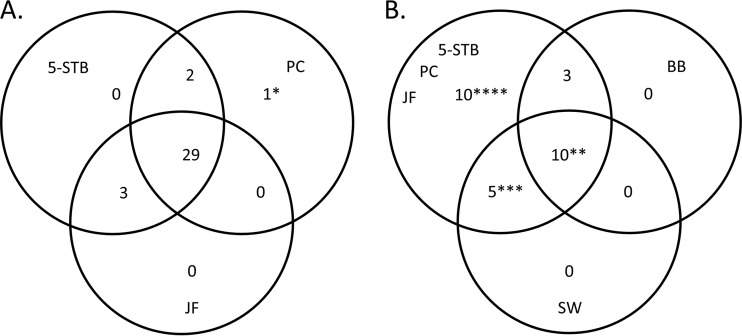
Contribution of specimen types to the diagnosis of prosthetic joint infection. (A) Thirty-five cases were diagnosed based on positive cultures of the minimum set comprising tissue biopsy specimens (5-STB), prosthetic components (PC), and joint fluid (JF). (B) A subgroup of 28 cases was evaluated based on all specimen types. It is apparent that neither the bone biopsy specimen (BB) nor the prosthetic swab (*in situ*) (SW) culture contributed independently to the diagnosis. *, one case diagnosis was supported by next-generation sequencing; **, one case showed a negative result in the culture of joint fluid; ***, one case showed negative results in the culture of prosthetic components; ****, in four cases, only one or two specimen types contributed to the culture-positive findings.

Positive cultures were obtained in a total of 39 cases. In all except four cases, the contributions of JF and PC project specimens could be evaluated at the same time as those of the 5-STB set (Fig. 4A). The three specimen types were sufficient for a PJI diagnosis in this subset of patients. In 28 revisions, all specimen types were collected (Fig. 4B). However, positive results from BB and SW specimens did not add any information to that gained from the JF, 5-STB, and PC set results. Of note, in one case, a positive culture was obtained only from the PC culture, and this finding was corroborated by 16S rRNA sequencing (Table S2).

## DISCUSSION

In this study, we found that results from JF, PC, and 5-STB set cultures were optimal for the diagnosis of PJI. Conversely, novel specimen types, including the BB acquired in close proximity to the prosthesis and the SW acquired from the prosthesis *in situ*, did not contribute independently to the diagnosis. The diagnostic contribution of specimen types has been addressed in several previous studies but has mostly focused on one diagnostic method or modification (20, 21, 34–38). Two recently published international guidelines for diagnosing PJI recommended the use of three to six biopsy specimens for culture without further specifications, and PCs were mentioned as an adjunct to the diagnosis (8, 9). In 2014, a French group reported that four specimens, independent of type, were the optimal number for diagnosing PJI from culture results (34). That study acknowledged a concern about cost containment, based on the likely rise in the number of revision surgeries, due to increasing numbers and longevity of patients with knee and hip arthroplasties (39). The French group also concluded that PCR-based methods had little diagnostic impact in the clinical setting (34, 35). To our knowledge, no previous study has compared combinations of specimen types and methods with the aim of identifying the most efficient and comprehensive (i.e., optimal) method for diagnosing PJI.

The heterogeneous group of patients in our study reflected clinical reality and lent strength to the results. Our prospective study design and diagnostic algorithm were important, because they provided a more uniform patient pathway than is otherwise possible. Nonetheless, our approach was flexible in accommodating the individual preferences of patients and surgeons. Both surgeons and nurses complied with the systematic sampling procedure during the entire study period (28). This consistency allowed direct comparisons of different specimen types and culture-based versus molecular analyses. A limitation of our study was the lack of an independent diagnostic reference. We accepted that the surgeons' initial diagnosis could be modified according to results from culturing the 5-STB set for 6 days and subsequent culturing results from project specimens according to the MSIS criteria from 2011 (31). Consequently, six diagnoses shifted, in one case from PJI to AF and in five cases from AF to PJI. These modest numbers emphasized the accuracy of the surgeons' initial judgment. Nevertheless, three cases lacked a definite diagnosis (one suspected of AF and two suspected of PJI), and we chose to exclude them from the final assessment.

Due to these limitations, statistical evaluation of diagnostic performance must be done cautiously. By calculating LR+ and LR− for different combinations of specimens and laboratory methods, we have provided an indication of the relative performance (Table 1). It is a concern that the diagnosis of PJI was mainly driven by culture; however, the diagnostic process was multifaceted, and the prevalence of PJI should not have a major impact on LR+ and LR−.

In the majority of cases, the use of antibiotics prior to sampling did not influence the results from culturing. Only two cases were identified as positive based on 16S rRNA sequencing alone (one had been diagnosed as PJI based on clinical indications, and one had remained undetermined). In both cases, sequencing revealed Streptococcus sp. infections.

Among the cases finally classified as PJI, we found a distribution of species that was different from those found in several other studies (7, 14, 36). This difference was probably due to the fact that 82% of our culture-positive cases were late infections (>12 weeks after primary surgery), including acute hematogenous infections and long-term chronic infections (Table S2). It is well known that the main pathogens in acute hematogenous infections are streptococci, enterococci, and S. aureus, and that chronic infections are mainly caused by CoNS (40), consistent with the distribution pattern observed in this study.

When we increased the incubation period from 6 to 14 days, we found a higher percentage of positives in the individual specimen types, in accordance with other studies (36, 38). However, when considering all specimens together in the incubation period of 6 days, the JF and PC cultures alone were positive in five cases (12%; 5/42); thus, they added significant information to the 31 cases identified with a positive 5-STB set. In three other cases (3/42; 7%), the JF and PC cultures were positive after 6 days of incubation. Still, three culture-negative cases were diagnosed based only on clinical parameters. Overall, these findings indicated that an additional 19% of PJI cases (8/42) could be confirmed with the JF, PC, and 5-STB set cultures (the optimal specimen set) and an incubation period of not less than 14 days (26) (Fig. 4A). This result suggested that a broad selection of specimens would permit shorter incubation times; nevertheless, 7% of PJIs could be missed. The number of cases in our study was too small to draw a definite conclusion, but we observed a trend.

For infections with an early, acute onset and rapidly growing bacteria (e.g., S. aureus, Streptococcus spp., and E. faecalis), all specimens may show positivity on day one, and microscopic analysis of JF specimens might reveal the infecting microorganism. The benefit of having a broad specimen collection was more apparent in difficult cases with low-grade infections (e.g., S. epidermidis and P. acnes) and in atypical and chronic cases. Classifying patients into those groups might facilitate allocating resources to patients most likely to benefit from extensive sampling and diagnostic work-up.

Comparing culturing and 16S rRNA sequencing approaches showed that sequencing only added a few extra findings. The optimization of culturing procedures, including specimen types, culture media, and incubation times, seemed to reduce the contribution of sequencing. Sequencing is costly and time-consuming, and it requires molecular expertise. Consequently, for economic reasons, its use may be restricted to cases where sequencing is critical, e.g., long-term chronic cases and cases with prior antibiotic administration. In general, the diagnostic contribution from 16S rRNA sequencing has been restricted to liquid specimens; this constraint might be due to the higher ratio of human to bacterial DNA in tissue biopsy specimens compared to JF and PC sonication fluid specimens (14, 35).

Future research regarding PJI diagnosis should target the difficult-to-diagnose cases in a prospective study. With the diagnostic specimen set concept, other promising culture and molecular methods can be tested; for example, automatic blood culture systems might be useful for some specimen types (20, 41, 42), and molecular methods might be useful for selected specimens from carefully defined patients. To show real diagnostic value, the contributions of different specimens must be evaluated in the context of difficult-to-diagnose cases. The overall goal must be a sensitive, specific, rapid diagnostic procedure that is readily applicable both for the surgeons and in the clinical microbiological routine.

## Supplementary Material

Supplemental material

## References

[1] Kamme C, Lindberg L 1981 Aerobic and anaerobic bacteria in deep infections after total hip arthroplasty: differential diagnosis between infectious and non-infectious loosening. Clin Orthop Relat Res 154:201–207. 7009009

[2] Gundtoft PH, Overgaard S, Schønheyder HC, Møller JK, Kjærgaard-Andersen P, Pedersen AB 2015 The “true” incidence of surgically treated deep prosthetic joint infection after 32,896 primary total hip arthroplasties. Acta Orthop 86:326–334. doi:10.3109/17453674.2015.1011983. 25637247 PMC4443464

[3] Atkins B, Athanasou N, Deeks JJ, Crook DWM, Simpson H, Peto TEA, McLardy-Smith P, Berendt AR, The Osiris Collaborative Study Group. 1998 Prospective evaluation of criteria for microbiological diagnosis of prosthesis joint infection at revision arthroplasty. J Clin Microbiol 36:2932–2939. 9738046 10.1128/jcm.36.10.2932-2939.1998 PMC105090

[4] DeHaan A, Huff T, Schabel K, Doung Y-C, Hayden J, Barnes P 2013 Multiple cultures and extended incubation for hip and knee arthroplasty revision: impact on clinical care. J Arthroplasty 28:59–65. doi:10.1016/j.arth.2013.03.037. 23886405

[5] Tunney MM, Patrick S, Gorman SP, Nixon JR, Anderson N, Davis RI, Hanna D, Ramage G 1998 Improved detection of infection in hip replacements. A currently underestimated problem. J Bone Joint Surg Br 80:568–572. doi:10.1302/0301-620X.80B4.8473. 9699813

[6] Trampuz A, Piper KE, Hanssen AD, Osmon DR, Cockerill FR, Steckelberg JM, Patel R 2006 Sonication of explanted prosthetic components in bags for diagnosis of prosthetic joint infection is associated with risk of contamination. J Clin Microbiol 44:628–631. doi:10.1128/JCM.44.2.628-631.2006. 16455930 PMC1392705

[7] Trampuz A, Piper KE, Jacobson MJ, Hanssen A, Unni KK, Osmon DR, Mandrekar J, Cockerill FR, Steckelberg JM, Geenleaf JF, Patel R 2007 Sonication of removed hip and knee prostheses for diagnosis of infection. N Engl J Med 357:654–663. doi:10.1056/NEJMoa061588. 17699815

[8] Høiby N, Bjarnsholt T, Moser C, Bassi GL, Coenye T, Donelli G, Hall-Stoodley L, Hola V, Imbert C, Kirketerp-Møller K, Lebeaux D, Oliver A, Ullmann AJ, Williams C, ESCMID Study Group for Biofilms and Consulting External Expert Werner Zimmerli. 2015 ESCMID guideline for the diagnosis and treatment of biofilm infections 2014. Clin Microbiol Infect 21:S1–S25. doi:10.1016/j.cmi.2014.10.024. 25596784

[9] Osmon DR, Berbari EF, Berendt AR, Lew D, Zimmerli W, Steckelberg JM, Rao N, Hanssen A, Wilson WR, Infectious Diseases Society of America. 2013 Diagnosis and management of prosthetic joint infection: clinical practice guidelines by the Infectious Diseases Society of America. Clin Infect Dis 56:e1–e25. doi:10.1093/cid/cis803. 23223583

[10] McDowell A, Patrick S 2005 Evaluation of nonculture methods for the detection of prosthetic hip biofilms. Clin Orthop Relat Res 437:74–82. 10.1097/01.blo.0000175123.58428.93 16056029

[11] Moojen DJ, Spijkers SN, Schot CS, Nijhof MW, Vogely HC, Fleer A, Verbout AJ, Castelein RM, Dhert WJ, Schouls LM 2007 Identification of orthopaedic infections using broad-range polymerase chain reaction and reverse line blot hybridization. J Bone Joint Surg Am 89:1298–1305. doi:10.2106/JBJS.F.00822. 17545434

[12] Tunney MM, Patrick S, Curran MD, Ramage G, Hanna D, Nixon JR, Gorman SP, Davis RI, Anderson N 1999 Detection of prosthetic hip infection at revision arthroplasty by immunofluorescence microscopy and PCR amplification of the bacterial 16S rRNA gene. J Clin Microbiol 37:3281–3290. 10488193 10.1128/jcm.37.10.3281-3290.1999 PMC85548

[13] Cazanave C, Greenwood-Quaintance KE, Hanssen A, Karau MJ, Schmidt SM, Urena EOG, Mandrekar J, Osmon DR, Lough LE, Pritt BS, Steckelberg JM, Patel R 2013 Rapid molecular microbiologic diagnosis of prosthetic joint infection. J Clin Microbiol 51:2280–2287. doi:10.1128/JCM.00335-13. 23658273 PMC3697690

[14] Ryu SY, Greenwood-Quaintance KE, Hanssen AD, Mandrekar JN, Patel R 2014 Low sensitivity of periprosthetic tissue PCR for prosthetic knee infection diagnosis. Diagn Microbiol Infect Dis 79:448–453. doi:10.1016/j.diagmicrobio.2014.03.021. 24972853

[15] Nolte FS 2015 Molecular microbiology, p 54–90. *In* Pfaller MA, Richter SS, Funke G, Jorgensen JH, Landry ML, Carroll KC, Warnock DW (ed), Manual of clinical microbiology, 11th ed ASM Press, Washington DC.

[16] Wolk DM, Dunne WM 2011 New technologies in clinical microbiology. J Clin Microbiol 49:S62–S67. doi:10.1128/JCM.00834-11.

[17] Bjerkan G, Witso E, Bergh K 2009 Sonication is superior to scraping for retrieval of bacteria in biofilm on titanium and steel surfaces in vitro. Acta Orthop 80:245–250. doi:10.3109/17453670902947457. 19404811 PMC2823171

[18] Cuñé J, Soriano A, Martinez JC, Garcia S, Mensa J 2009 A superficial swab culture is useful for microbiologic diagnosis in acute prosthetic joint infections. Clin Orthop Relat Res 467:531–535. doi:10.1007/s11999-008-0553-4. 18850254 PMC2628497

[19] Esteban J, Gomez-Barrena E, Cordero J, Martin-de-Hijas NZ, Kinnari TJ, Fernandez-Roblas R 2008 Evaluation of quantitative analysis of cultures from sonicated retrieved orthopedic implants in diagnosis of orthopedic infection. J Clin Microbiol 46:488–492. doi:10.1128/JCM.01762-07. 18077647 PMC2238112

[20] Font-Vizcarra L, Garcia S, Martinez-Pastor JC, Sierra JM, Soriano A 2010 Blood culture flasks for culturing synovial fluid in prosthetic joint infections. Clin Orthop Relat Res 468:2238–2243. doi:10.1007/s11999-010-1254-3. 20162386 PMC2895826

[21] Gallo J, Kolar M, Dendis M, Loveckova Y, Sauer P, Zapletalova J, Koukalova D 2008 Culture and PCR analysis of joint fluid in the diagnosis of prosthetic joint infection. New Microbiol 31:97–104. 18437847

[22] Levine BR, Evans BG 2001 Use of blood culture vial specimens in intraoperative detection of infection. Clin Orthop Relat Res 382:222–231. doi:10.1097/00003086-200101000-00030. 11153992

[23] Piper KE, Jacobson MJ, Cofield RH, Sperling JW, Sanchez-Sotelo J, Osmon DR, McDowell A, Patrick S, Steckelberg JM, Mandrekar JN, Fernandez Sampedro M, Patel R 2009 Microbiologic diagnosis of prosthetic shoulder infection by use of implant sonication. J Clin Microbiol 47:1878–1884. doi:10.1128/JCM.01686-08. 19261785 PMC2691098

[24] Larsen LH, Lange J, Xu Y, Schønheyder HC 2012 Optimizing culture methods for diagnosis of prosthetic joint infections: a summary of modifications and improvements reported since 1995. J Med Microbiol 61:309–316. doi:10.1099/jmm.0.035303-0. 22222201

[25] Larsen LH, Xu Y, Pedersen M, Schønheyder HC, Thomsen TR 2014 Long-term storage of clinical samples in CyMol medium for PNA-FISH and culturing from the eSwab system. Abstr. 24th Eur Cong Clin Microbiol Infect Dis, abstr P0606.

[26] Larsen LH, Xu Y, Khalid V, Aleksyniene R, Thomsen TR, Schønheyder HC 2015 The optimal set for culture-based diagnosis of prosthetic joint infections in the hip or knee. Abstr 25th Eur Cong Clin Microbiol Infect Dis, abstr P1356.

[27] Khalid V, Larsen LH, Schønheyder HC, Thomsen TR, Lorenzen J, Aleksyniene R, Frost M, Rasmussen S, Study Group PRIS. 2014 New diagnostic algorithm in evaluation of patients with prosthesis related problems in the hip or knee. Abstr 33rd Annu Meet Eur Bone Joint Infect Soc, abstr F005.

[28] Larsen LH, Xu Y, Simonsen O, Pedersen C, Schønheyder HC, Thomsen TR, Study Group PRIS. 2014 “All in a box” a concept for optimizing microbiological diagnostic sampling in prosthetic joint infections. BMC Res Notes 7:418. doi:10.1186/1756-0500-7-418. 24993888 PMC4105167

[29] Xu Y, Rudkjøbing VB, Simonsen O, Pedersen C, Lorenzen J, Schønheyder HC, Nielsen PH, Thomsen TR 2012 Bacterial diversity in suspected prosthetic joint infections: an exploratory study using 16S rRNA gene analysis. FEMS Immunol Med Microbiol 65:291–304. doi:10.1111/j.1574-695X.2012.00949.x. 22364231

[30] McIlroy SJ, Saunders AM, Albertsen M, Nierychlo M, McIlroy B, Hansen AA, Karst SM, Nielsen JL, Nielsen PH 2015 MiDAS: the field guide to the microbes of activated sludge. Database (Oxford) 2015:bav062. doi:10.1093/database/bav062. 26120139 PMC4483311

[31] Parvizi J, Zmistowski B, Berbari EF, Bauer TW, Springer BD, Della Valle CJ, Garvin KL, Mont MA, Wongworawat MD, Zalavras CG 2011 New definition for periprosthetic joint infection: from the Workgroup of the Musculoskeletal Infection Society. Clinl Orthop Relat Res 469:2992–2994. doi:10.1007/s11999-011-2102-9. PMC3183178 21938532

[32] Guyatt GD, Sackett DL, Haynes RB 2006 Evaluating diagnostic test, p 273–322. *In* Tugwell P, Sackett DL, Guyatt GD, Haynes RB (ed), Clinical epidemiology: how to do clinical practice research, 2nd ed Lippincott Williams & Wilkins, Philadelphia, PA.

[33] McGee S 2002 Simplifying likelihood ratios. J Gen Intern Med 17:646–649. doi:10.1046/j.1525-1497.2002.10750.x. 12213147 PMC1495095

[34] Bémer P, Léger J, Tandé D, Plouzeau C, Valentin AS, Jolivet-Gougeon A, Lemarié C, Kempf M, Héry-Arnaud G, Bret L, Juvin ME, Giraudeau B, Corvec S, Burucoa C, Centre de Référence des Infections Ostéo-Articulaires du Grand Ouest (CRIOGO) Study Team. 2016 How many samples and how many culture media to diagnose a prosthetic joint infection: a clinical and microbiological prospective multicenter study. J Clin Microbiol 54:385–391. doi:10.1128/JCM.02497-15. 26637380 PMC4733176

[35] Bémer P, Plouzeau C, Tande D, Leger J, Giraudeau B, Valentin AS, Jolivet-Gougeon A, Vincent P, Corvec S, Gibaud S, Juvin ME, Hery-Arnaud G, Lemarie C, Kempf M, Bret L, Quentin R, Coffre C, de Pinieux G, Bernard L, Burucoa C, Centre de Référence des Infections Ostéo-Articulaires du Grand Ouest (CRIOGO) Study Team. 2014 Evaluation of 16S rRNA gene PCR sensitivity and specificity for diagnosis of prosthetic joint infection: a prospective multicenter cross-sectional study. J Clin Microbiol 52:3583–3589. doi:10.1128/JCM.01459-14. 25056331 PMC4187742

[36] Butler-Wu SM, Burns EM, Pottinger PS, Magaret AS, Rakeman JL, Matsen FA III, Cookson BT 2011 Optimization of periprosthetic culture for diagnosis of Propionibacterium acnes prosthetic joint infection. J Clin Microbiol 49:2490–2495. doi:10.1128/JCM.00450-11. 21543562 PMC3147880

[37] Hughes JG, Vetter EA, Patel R, Schleck CD, Harmsen S, Turgeant LT, Cockerill FR 2001 Culture with BACTEC Peds Plus/F bottle compared with conventional methods for detection of bacteria in synovial fluid. J Clin Microbiol 39:4468–4471. doi:10.1128/JCM.39.12.4468-4471.2001. 11724863 PMC88567

[38] Schäfer P, Fink B, Sandow D, Margull A, Berger I, Frommelt L 2008 Prolonged bacterial culture to identify late periprosthetic joint infection: a promising strategy. Clin Infect Dis 47:1403–1409. doi:10.1086/592973. 18937579

[39] Kurtz SM, Lau E, Watson H, Schmier JK, Parvizi J 2012 Economic burden of periprosthetic joint infection in the United States. J Arthroplasty 27:61–65.e1. doi:10.1016/j.arth.2012.02.022. 22554729

[40] Stefansdottir A, Johansson D, Knutson K, Lidgren L, Robertsson O 2009 Microbiology of the infected knee arthroplasty: report from the Swedish Knee Arthroplasty Register on 426 surgically revised cases. Scand J Infect Dis 41:831–840. doi:10.3109/00365540903186207. 19922065

[41] Peel TN, Dylla BL, Hughes JG, Lynch DT, Greenwood-Quaintance KE, Cheng AC, Mandrekar JN, Patel R 2016 Improved diagnosis of prosthetic joint infection by culturing periprosthetic tissue specimens in blood culture bottles. mBio 7:e01776-15. doi:10.1128/mBio.01776-15. 26733067 PMC4725002

[42] Peel TN, Sedarski JA, Dylla BL, Shannon SK, Amirahmadi F, Hughes JG, Cheng AC, Patel R 2017 Laboratory workflow analysis of culture of periprosthetic tissues in blood culture bottles. J Clin Microbiol 55:2817–2826. doi:10.1128/JCM.00652-17. 28701418 PMC5648717

