# Supplementary material for: Differential Contributions of Specimen Types, Culturing, and 16S rRNA Sequencing in Diagnosis of Prosthetic Joint Infections
Source: J Clin Microbiol. 2018 Apr 25;56(5):e01351-17. doi: 10.1128/JCM.01351-17 (PMC5925708; doi:10.1128/JCM.01351-17)
Supplement: Supplemental material [file supp_56_5_e01351-17__index.html]

Differential Contributions of Specimen Types, Culturing, and 16S rRNA Sequencing in Diagnosis of Prosthetic Joint Infections — Supplemental material 

# Differential Contributions of Specimen Types, Culturing, and 16S rRNA Sequencing in Diagnosis of Prosthetic Joint Infections

## Supplemental material

- Supplemental file 1 -

  Text S1 (Detailed culture methods)

  PDF, 193K
- Supplemental file 2 -

  Table S2 (Overview of culturing and molecular data)

  XLSX, 23K
- Supplemental file 3 -

  Text S3 (Background subtraction in 16S amplicon data analysis)

  PDF, 85K
