# Supplementary material for: Differential Contributions of Specimen Types, Culturing, and 16S rRNA Sequencing in Diagnosis of Prosthetic Joint Infections
Source: J Clin Microbiol. 2018 Apr 25;56(5):e01351-17. doi: 10.1128/JCM.01351-17 (PMC5925708; doi:10.1128/JCM.01351-17)
Supplement: Supplemental material [file JCM.01351-17_zjm999095913s1.pdf]

Supplementary material S1

Detailed culture methods

Culture was processed according to local guidelines which are inoculating onto 5% horse blood agar, chocolate agar, and chocolate agar (+fosfomycin[200µg]) and incubated aerobic in 5% CO_2 (SSI Diagnostika, Denmark). Chocolate agar enforced with vitamin K (+metronidazole [5 µg], +kanamycin [500 µg]) and 10% horse blood agar (+metronidazole [5 µg], +kanamycin [500 µg]) for anaerobic culture (SSI Diagnostika, Denmark). Thioglycollat plus 10% glycerol and serum broth (SSI Diagnostika, Denmark) and ChromID™ CPS® Elite (BioMérieux, France) incubated aerobe.

A light microscopy was performed directly on joint fluid together with a Gram-stained sample before culturing without further processing. The tissue biopsies were cut into pieces corresponding to the amount of plates/media used, using separate forceps and scalpel. Two imprints were made on the agar plate, one being streaked. Only one of the five biopsies were inoculated on all media, the remaining 4 were inoculated only aerobic on 5% horse blood agar, chocolate agar and anaerobic on chocolate agar enforced with vitamin K, with selective antibiotics as above.

All culture were incubated for 14 days; aerobic media were evaluated at days 1, 2, 4, 6, 10, and 14 and anaerobic media at days 2, 4, 6, 10, and 14. If the cultures were positive at day 6, subcultures were inoculated from thioglycolat at 5% horse blood agar, chocolate agar (+fosfomycin[200 µg]) and chocolate agar enforced with vitamin K (+metronidazole [5 µg], +kanamycin [500 µg]) and incubated as above for 4 days, to find more fastidious bacteria there might have been overgrowth on the primary plate. If the primary cultures were negative at day 10, identical subcultures from the thioglycolat made without selective antibiotics and incubated for 4 days.
