# Supplementary material for: Differential Contributions of Specimen Types, Culturing, and 16S rRNA Sequencing in Diagnosis of Prosthetic Joint Infections
Source: J Clin Microbiol. 2018 Apr 25;56(5):e01351-17. doi: 10.1128/JCM.01351-17 (PMC5925708; doi:10.1128/JCM.01351-17)
Supplement: Supplemental material [file JCM.01351-17_zjm999095913s3.pdf]

S3

Background subtraction in 16S amplicon data analysis

DNA extraction negative controls and PCR negative controls were included in every batch analysis (in total > 50 controls). Most of the sequences/species regularly found in these negative controls were normally considered as environment species. Therefore we consider these species as background. In addition, we previously performed 16S *rRNA* amplicon sequencing on serial dilutions of two pure cultures spiked with sterile human joint fluid (one *Enterococcus phoeniculicola* and the other *Salmonella bongori*), all the species other than *E. phoeniculicola* and *S. bongori* were considered as background (the results are not included in this study). Furthermore, some common skin bacteria (*Staphylococcus* species, *Propionibacterium* spp., *Pseudomonas* spp. etc.) were frequently detected at very low numbers (maximum a few hundred reads/specimen) in the negative controls. We consider these as background because a true infection by these species gives a lot more sequences according to our experience.

Therefore, in general we remove all OTUs with less than 200 reads, and then all OTUs which are known environmental contaminants. Lastly the OTUs with more than 1000 reads were considered as true pathogen.
